# Restoration of multi-channel signal loss using autoencoder with recursive input strategy

**DOI:** 10.1038/s41598-025-98374-5

**Published:** 2025-04-21

**Authors:** Jaejun Lee, Yonggyun Yu, Hogeon Seo

**Affiliations:** 1 Korea Atomic Energy Research Institute, 111, Daedeok-daero 989beon-gil, Yuseong-gu, Daejeon 34057 Republic of Korea; 2 University of Science & Technology, 217, Gajeong-ro, Yuseong-gu, Daejeon 34113 Republic of Korea

**Keywords:** Data restoration, Autoencoder, Recursive input strategy, Dynamic termination, Mechanical engineering, Computer science, Software, Information technology

## Abstract

Multi-channel sensor data often suffer from missing or corrupted values due to sensor failures, communication disruptions, or environmental interference. These issues severely limit the accuracy of intelligent systems relying on sensor data integration. Existing data restoration techniques often fail to capture complex correlations among sensor channels, especially when data losses occur randomly and continuously. To overcome these limitations, we propose an autoencoder-based data recovery algorithm that recursively feeds reconstructed outputs back into the model to progressively refine estimates. A dynamic termination criterion monitors reconstruction improvements, automatically stopping iterations when further refinements become negligible. This recursive input strategy significantly enhances restoration accuracy and computational efficiency compared to conventional single-step methods. Experiments on multivariate sensor datasets show that the proposed method significantly outperforms the one-time autoencoder restoration method and maintains robust performance across diverse datasets and missing data scenarios. This approach provides a scalable and adaptable solution to ensure data integrity in complex sensor networks, enabling improved reliability and operational efficiency in industrial and technological applications.

## Introduction

The contemporary industrial and technological sectors are undergoing rapid changes driven by advances in automation and the development of intelligent systems. In these systems, sensors provide crucial data to support various operational and decision-making processes^1^. For example, sensors monitor the condition of machinery in manufacturing processes, enabling predictive maintenance^2^. In smart cities, sensors are employed to track traffic and energy consumption in real-time, facilitating efficient urban management^3^. The integration of data from multiple sensors is paramount for comprehending system states and environmental conditions. In environmental monitoring, data on air quality, weather patterns, and pollutant levels are collected from various locations to inform public health decisions and environmental policies^4^. In the healthcare sector, the integration of wearable sensors has emerged as a pivotal element in the continuous monitoring of physiological signals, including heart rate, blood pressure, and oxygen saturation. This integration facilitates the real-time tracking of patients’ health conditions, enabling early diagnosis and personalized treatment through the integration of data^5^. The integration of data from various sensors is also crucial in autonomous vehicles. The fusion of data from sensors such as cameras, LiDAR, radar, and ultrasonic sensors enables the accurate perception of the surrounding environment and ensures safe navigation^6^.

However, the increasing reliance on sensor data underscores the imperative for ensuring its reliability and completeness, as the entire system’s integrity may be compromised if data from any sensor in the network is missing or corrupted. This can result in degraded performance or erroneous decisions. For instance, hardware failures can incapacitate a sensor’s ability to collect data, while signal loss due to communication disruptions or battery depletion can affect data transmission^7^. Environmental factors, including extreme temperatures, humidity changes, and electromagnetic interference, can also degrade the accuracy and reliability of sensors^8^. The absence of data from certain sensors can have a substantial impact on data fusion processes that rely on integrating data from multiple sources. In structural health monitoring (SHM), integrating data from various sensors is imperative for accurately assessing the condition of infrastructure, including bridges and buildings. The absence of data from specific sensors may impede the identification of minor cracks or deformations, which could ultimately result in catastrophic failures^9^. In the context of autonomous vehicles, the loss of data from vital sensors, such as LiDAR or radar, can hinder the vehicle’s capacity to detect objects and avoid collisions^10^. In healthcare, the absence of heart rate data during cardiac monitoring may impede the detection of life-threatening arrhythmias^11^.

To address the aforementioned challenges, there is a necessity for effective data restoration methods to reconstruct missing or corrupted sensor data. Ensuring the completeness and reliability of the data is instrumental in facilitating efficient system operations, enhancing the performance of intelligent algorithms, and minimizing errors in decision-making processes^12^. However, traditional data restoration techniques, such as mean imputation, k-nearest neighbors, and regression analysis, often struggle to capture the complex correlations present in multivariate sensor datasets^13^. These conventional techniques necessitate exhaustive information about the data both before and after the missing values, impeding the utilization of intricate interdependencies among different sensors. Recent advancements in machine learning and deep learning have demonstrated the potential to overcome certain limitations of traditional methods. Nevertheless, these novel approaches encounter challenges as well. Their efficacy can be diminished when confronted with irregular or continuous missing data patterns, and as complexity increases, automation can become more arduous^14,15^.

To overcome these challenges, we propose a data restoration algorithm utilizing an autoencoder with a recursive input strategy. This algorithm employs an autoencoder, thereby enabling the method to restore missing values by leveraging the relationships and information between different channels or sensor data, even at the current time point. To achieve more complete restoration, a recursive strategy is applied that feeds the output back as input, allowing for iterative refinement. Additionally, automation and flexibility are enhanced by dynamically adapting the termination of the recursive process to match changing data characteristics. To validate the effectiveness of the proposed algorithm, we employed multivariate sensor data commonly used in various industrial and technological applications. This demonstrates that the proposed method can effectively solve data loss problems occurring in complex sensor networks and enhance the system’s reliability and performance. Through experimentation with real-world datasets, we have validated that our method effectively restores missing or corrupted data without the need for prior knowledge of missing data patterns, and adapts to various data distributions and missing value scenarios.

The remainder of this paper is organized as follows. In the section of Related work, a review of related data restoration methods and their applications across different fields is presented, along with a discussion of the limitations of the approach under consideration. In “Methods” section, the proposed methodology is described in detail, including the autoencoder architecture, the recursive input strategy, and the dynamic stopping criterion. “Experiments” section outlines the experimental setup and results, demonstrating the effectiveness of the proposed method. The final section, “Discussion” section, offers a conclusion to the paper and puts forward potential avenues for future research.

## Related works

Conventional restoration techniques, encompassing statistical methods and interpolation techniques like linear, spline, and nearest neighbor interpolation, are prevalent due to their accessibility. These techniques are effective for univariate data with simple patterns; however, they frequently encounter limitations in complex, nonlinear, or dynamic multivariate scenarios characteristic of contemporary sensor networks. These conventional methods frequently encounter difficulties in discerning intricate dependencies or adapting to data shifts, which can result in inaccurate estimations^16–18^. Furthermore, techniques such as mean or regression imputation have been observed to introduce bias and underestimate variability, thereby compromising data reliability^19^. Machine learning techniques have been employed with increasing frequency to address the limitations of traditional methods, primarily by learning from data to capture complex relationships and dependencies among variables. Methods such as k-nearest neighbors (k-NN), support vector machines (SVM), and decision trees have been utilized for imputing missing data^20,21^. In particular, deep learning methods show significant promise due to their capability to model nonlinear relationships and handle high-dimensional data. The application of recurrent neural networks (RNNs) and long short-term memory (LSTM) networks have been instrumental in the analysis of temporal dependencies in time-series sensor data, while convolutional neural networks (CNNs) have been leveraged to exploit spatial correlations in sensor networks^22,23^.

Autoencoders, a category of unsupervised deep learning models, have demonstrated remarkable efficacy in tasks involving data restoration due to their capacity to learn efficient data representations. These models comprise two primary components: an encoder, which compresses the input data into a latent space, and a decoder, which reconstructs the data from this compressed representation. Through training on extensive datasets, autoencoders develop a profound comprehension of the data’s underlying structure^24^. In the context of missing data imputation, autoencoders exhibit a remarkable ability to predict missing values by reconstructing data from the latent space, leveraging the inherent correlations among variables. Numerous studies have validated the efficacy of autoencoders in restoring missing sensor data. For instance, Kascenas et al.^25^ employed denoising autoencoders to reconstruct medical data, surpassing conventional imputation methods. Furthermore, Chen et al.^26^ employed a variation of an encoder and decoder to impute missing data in environmental sensor networks, thereby enhancing the performance of air quality monitoring systems. Similarly, Ba-Alawi et al.^27^ utilized variational residual autoencoders to impute missing data in wastewater treatment plants, thus enhancing the performance of sustainable operation.

To enhance the precision of the restoration process, certain researchers have adopted recursive or iterative methodologies. These approaches involve the iterative refinement of the estimation of missing values by repeatedly introducing the output into the model. Zhou and Huang^28^ have proposed an interactive imputing network (IIN) based on long short-term memory (LSTM), which has exhibited resilience to diverse missing scenarios by progressively adapting missing values through the joint utilization of information from both visible observations and previously imputed missing values. Pan et al.^29^ have achieved substantial gains in learning efficiency through the recursive imputation of missing values during training, leveraging a deep autoencoder (DAE). However, many recursive methods depend on predetermined termination conditions to conclude within iterative processes. Additionally, the integration of incremental steps during the training phase has the potential to augment the complexity of the learning process, thereby impeding the model’s capacity to discern salient features from data effectively^30,31^. Consequently, this can diminish the model’s adaptability to novel data, characterized by divergent patterns.

In this study, we propose an autoencoder to restore missing values without reliance on temporal information, thereby providing the advantage of reconstruction without time dependencies. Furthermore, a recursive strategy is employed during the inference phase to achieve more complete data restoration. This strategy distinguishes the proposed approach from traditional methods, as it can operate independently of specific assumptions, conditions, or a particular neural network backbone, allowing seamless integration into existing systems. The model dynamically monitors changes during the recursive process, applying tailored termination conditions that enhance its adaptability and automation potential (Table 1).

**Table 1:** Comparison of missing data restoration methods: strengths, limitations, and references.

Method category	Strengths	Limitations	References
Statistical methods	Simple implementation, computationally efficient, easy interpretability	Poor handling of nonlinear, complex correlations, biased estimations	^16–19^
Machine learning	Capable of modeling nonlinearity, improved accuracy compared to simple methods	Require careful feature engineering, sensitive to data quality, limited scalability	^20,21^
Deep learning	Strong ability to model complex and nonlinear relationships, handle large-scale data	Computationally intensive, risk of overfitting, performance-sensitive to hyperparameters	^22–26^
Recursive/Iterative methods	Adaptive refinement, iterative accuracy improvement, robust to diverse missing scenarios	High computational cost, requires suitable stopping criteria, risk of error propagation	^28–31^
Proposed method (Recursive Autoencoder)	Iterative refinement without reliance on temporal dependencies, dynamic termination criterion ensures computational efficiency, robust across diverse missing patterns and scenarios	Relies primarily on inter-channel correlations, potentially limiting accuracy when correlations between sensor channels are weak	Ours

## Methods

**Fig. 1:**
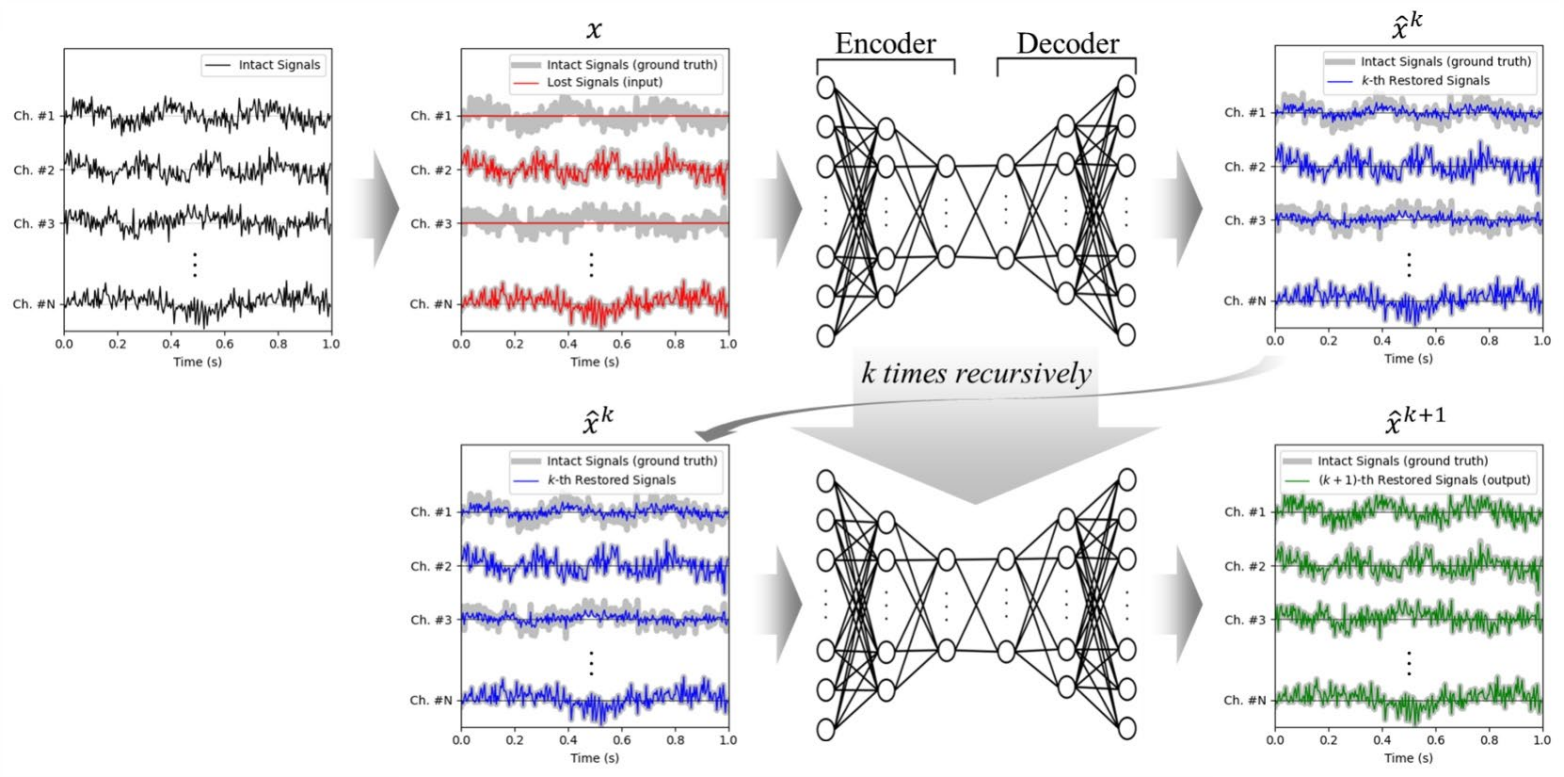
Structure of the proposed method utilizing recursive input strategy with dynamic termination criteria. The autoencoder refines the reconstruction iteratively until the stopping condition based on error monitoring is satisfied.

The methodology is predicated on the training of an autoencoder exclusively on normal data. Given a dataset ${\textbf{X}} \in {\mathbb {R}}^{N \times C}$ with missing values at specific positions, our objective is to restore these missing values without relying on temporal information or predefined assumptions about the missing data pattern. This approach stands in contrast to existing methods, such as Kalman filtering or autoregressive models, which require historical data, and Transformer-based imputation methods, which require extensive training on large datasets. Our method functions independently of temporal dependencies and can be seamlessly integrated into various neural architectures.

The employment of uncorrupted data exclusively during the training process enables the autoencoder to discern the intrinsic structures and patterns that define the normal data distribution. This training regimen ensures that the model attains proficiency in the reconstruction of inputs that align with the learned distribution while maintaining sensitivity to deviations indicative of corrupted or anomalous data. By excluding corrupted data during the training process, the model avoids the assimilation of incorrect representations, thereby preserving its reconstruction capabilities during inference. The reconstruction error is quantified using the Root Mean Square Error (RMSE), defined as follows:

1 $$\begin{aligned} L_{RMSE} = \sqrt{\frac{\sum _{i=1}^{N} \sum _{j=1}^{C} \left( X_{i,j} - {\hat{X}}_{i,j} \right) ^2}{NC}}, \end{aligned}$$

where $X_{i,j}$ represents the original data point, ${\hat{X}}_{i,j}$ denotes the reconstructed data point from the autoencoder, *N* signifies the total number of samples, and *C* is the number of features. The $L_{RMSE}$ penalizes significant deviations more heavily due to the squaring operation, thereby encouraging the model to minimize substantial reconstruction errors. Additionally, RMSE is continuous and differentiable, making it suitable for gradient-based optimization techniques commonly used in neural network training^32^.

### Recursive input strategy with dynamic termination

To iteratively refine the reconstruction of missing or corrupted values, a recursive input strategy is introduced during inference. Rather than passing the input through the autoencoder only once, we iteratively reintroduce the autoencoder’s output as input for further refinement, as illustrated in Fig. 1. The initial input consists of a dataset with missing or corrupted values, marked by a mask indicating their positions. At each iteration *k*, the autoencoder processes the current estimate ${\hat{X}}^{(k)}$ to produce an updated reconstruction ${\hat{X}}^{(k+1)}$, defined as:

2 $$\begin{aligned} {\hat{X}}^{(k+1)} = AE\left( {\hat{X}}^{(k)} \right) = D_\theta \left( E_\phi \left( {\hat{X}}^{(k)} \right) \right) , \end{aligned}$$

where *AE* denotes the autoencoder, consisting of an encoder $E_{\phi }$ and a decoder $D_{\theta }$ parameterized by $\phi$ and $\theta$, respectively. The recursion begins with the initial estimate:

3 $$\begin{aligned} {\hat{X}}^{(0)} = X \odot M + {\hat{X}}_{init} \odot (1 - M), \end{aligned}$$

where *M* is the binary mask indicating known values ($M_{i,j} = 1$) and missing values ($M_{i,j} = 0$), and ${\hat{X}}_{init}$ is an initial estimate for the missing values, which can be set using simple statistical imputation (e.g., mean imputation) or an informed prior. This recursive process enables the autoencoder to iteratively refine its estimations of the missing or corrupted values by leveraging its learned representation of the normal data distribution. Consequently, the reconstruction accuracy is enhanced over successive iterations, thereby improving the overall quality of the data. To determine an optimal stopping point for the recursion, a dynamic termination mechanism is implemented based on reconstruction error monitoring. This mechanism adapts to varying data characteristics and prevents unnecessary iterations. First, the change in the total reconstruction error across iterations is monitored using the following formula:

4 $$\begin{aligned} \Delta E^{(k)}=E^{(k-1)}-E^{(k)}. \end{aligned}$$

If the error has increased, as indicated by a negative value of $\Delta E^{(k)}$, the error increase counter, designated as $p_{inc}$, is incremented. If this counter reaches a predefined patience level, designated as *P*, the recursion is terminated to prevent further degradation. Secondly, the rate of improvement is evaluated using the relative error reduction, defined as follows:

5 $$\begin{aligned} r^{(k)} = \frac{|\Delta E^{(k)}|}{E^{(k-1)} + \epsilon }, \end{aligned}$$

In order to circumvent the division by zero, it is imperative to incorporate a negligible constant, designated as $\epsilon$. The moving average, denoted by ${\bar{r}}^{(k)}$, is computed over the last *W* iterations. If ${\bar{r}}^{(k)}$ falls below a minimum relative error reduction threshold, designated as $\epsilon _{dyn}$ for consecutive iterations exceeding *P*, it is deduced that further iterations will not yield substantial improvements, and the recursion is terminated. The dynamic threshold, denoted by $\epsilon _{dyn}$, is defined as a fraction $\alpha$ of the initial error $E^{(0)}$, allowing the termination criterion to adapt to varying error scales:

6 $$\begin{aligned} \epsilon _{dyn} = \alpha E^{(0)}. \end{aligned}$$

The parameters *W*, $\alpha$, and *P* have a significant impact on reconstruction efficiency and termination behavior. An increase in the window size *W* reduces variations in error reduction, but it may also result in a delay in termination. A small threshold ratio $\alpha$ ensures high reconstruction accuracy, while a suitable patience level *P* prevents premature stopping.

### Computational efficiency considerations

Contrary to the fixed-iteration methods, our approach utilizes dynamic adjustment of the number of recursive steps based on convergence criteria, thereby ensuring computational adaptability. The worst-case complexity of our approach is $O(K \cdot f(N, C))$, where *K* denotes the number of iterations and *f*(*N*, *C*) represents the computational cost of a single forward pass-through the autoencoder. However, due to the adaptive nature of recursion, the actual cost is frequently considerably lower, leading to a reduction in unnecessary computations. The integration of these termination criteria enhances reconstruction accuracy while optimizing computational efficiency. The recursive input strategy effectively reduces residual errors, and the termination mechanism prevents excessive iterations.

### Implementation details

**Algorithm 1:**
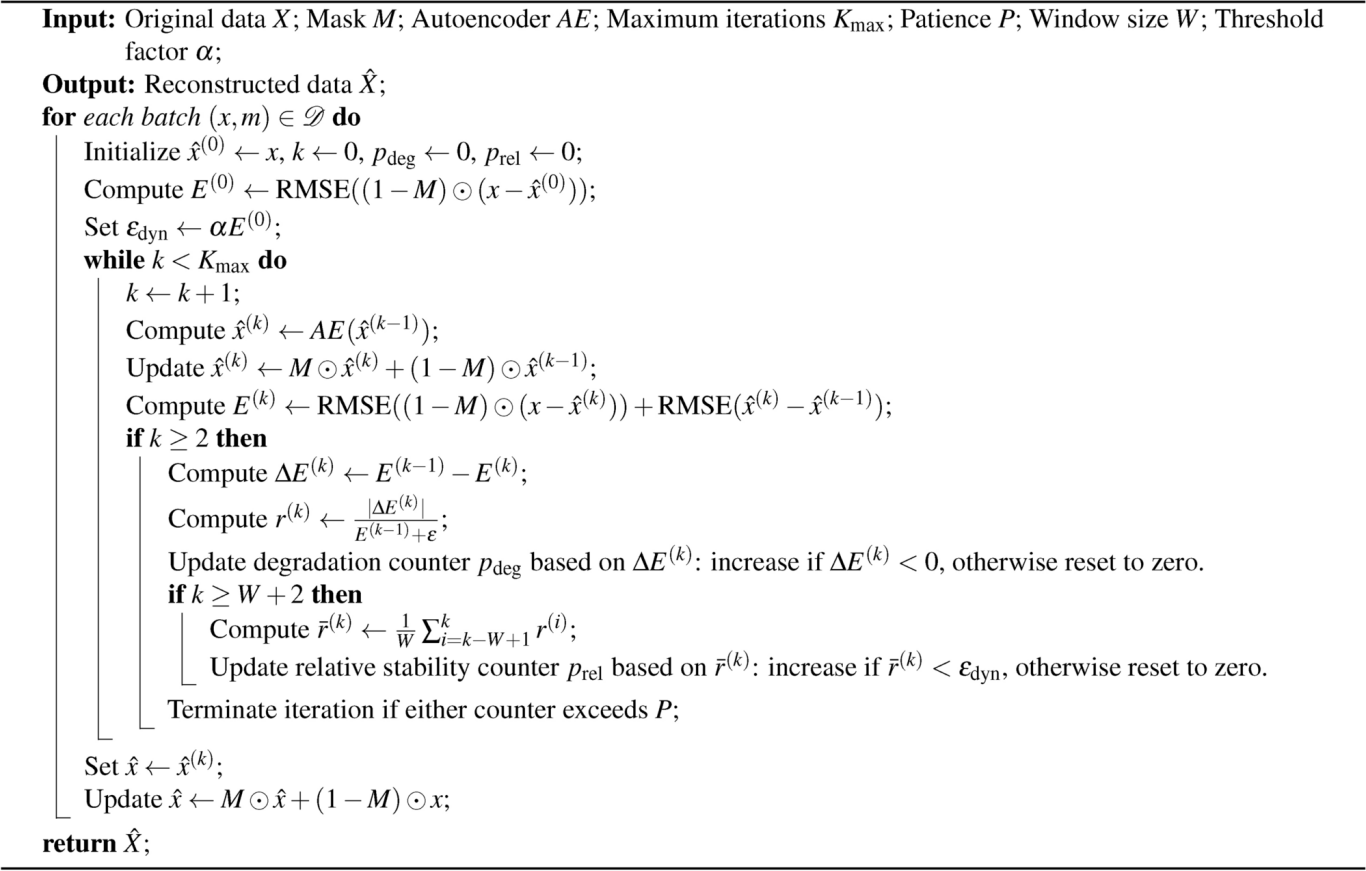
Recursive Inference with Dynamic Termination

Algorithm 1 delineates the recursive inference process. Initially, the autoencoder is trained on standard data. During the process of inference, the recursive input strategy is implemented, and termination is dynamically regulated using the error monitoring criteria previously outlined. Figure 1 provides a visual representation of the approach, illustrating how the autoencoder iteratively reconstructs missing or corrupted data until the dynamic termination conditions are met.

## Experiments

### Datasets

We perform experiments on several widely used public benchmark datasets, specifically the four ETT datasets (ETTh1, ETTh2, ETTm1, ETTm2)^33^ and the Weather dataset. All datasets consist of time-series data. Table 2 summarizes the key characteristics and statistics of these datasets. The ETT (Electricity Transformer Temperature) datasets were collected from two different counties in China between July 2016 and July 2018. ETTh1 and ETTh2 were recorded at hourly intervals, while ETTm1 and ETTm2 were recorded at 15-minute intervals. Each data point includes the target variable, oil temperature, and six power load features. The Weather dataset contains meteorological data recorded at 10-minute intervals throughout the year 2020. Each data point comprises 21 meteorological indicators, including air temperature, humidity, and air pressure.

**Table 2:** Characteristics and statistics of the utilized benchmark datasets.

Dataset	Channels	Data points	Granularity
ETTh1	7	17420	Hourly
ETTh2	7	17420	Hourly
ETTm1	7	69680	15 Minutes
ETTm2	7	69680	15 Minutes
Weather	21	52696	10 Minutes

### Experimental details

**Fig. 2:**
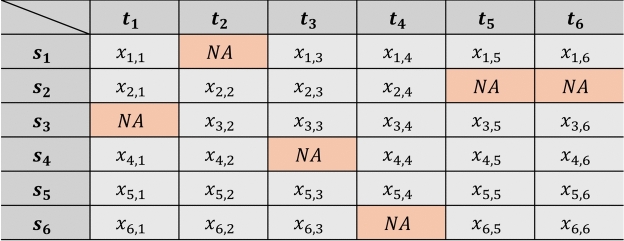
Illustration of a missing data scenario where certain data points are randomly masked, shown as NA.

In the present experiments, all channels from each dataset were utilized, except the target value channel. To simulate scenarios in which data is missing at random, a random points scenario was applied by replacing specific data points within channels with zeros, thereby effectively masking them, as illustrated in Fig. 2. This approach is predicated on the assumption that missing values are randomly distributed across different channels and time points. After the application of this masking, the data was scaled using a standard scaler individually on each channel. Given that the original missing values are substituted with zeros, and scaling centers the data to have a mean of zero and a variance of one, the masked values correspond to the mean of the transformed data distribution.

The utilization of data from disparate channels, each characterized by its own scale and distribution, has the potential to result in the model’s bias towards channels exhibiting larger scales. Furthermore, the comparison of data with distinct distributions can be challenging, a circumstance that can exert a deleterious effect on the model’s learning speed and performance. To address this issue, a solution is to scale each channel individually, thereby standardizing the data and facilitating easier comparison. This approach helps to prevent scale-related biases and enhance the model’s learning process, potentially improving its generalization performance. After transforming the data, 60% of the total data was used for training, while the remaining 40% was equally split between validation and testing, allocating 20% to each.

The autoencoder (AE) comprises two components: an encoder and a decoder, each with three layers. The number of features in the encoder’s hidden layers begins at 1024 and is reduced by half with each subsequent layer (i.e., 1024, 512, 256). Conversely, the decoder commences with 256 features and doubles the number of features with each successive layer (i.e., 256, 512, 1024). Additionally, we apply batch normalization (BN), the rectified linear unit (ReLU) activation function, and dropout with a probability of 25% are applied to all layers except the last layer in both the encoder and decoder. This strategy helps prevent overfitting, introduces non-linearity, and enhances training stability by normalizing inputs, adding activation functions, and randomly deactivating neurons during each training iteration. To investigate the effectiveness of different feature extraction mechanisms in modeling inter-channel relationships, we introduce two variants of the base AE architecture: AE-C, which incorporates a 1D CNN layer to capture local dependencies between channels, and AE-A, which integrates a multi-head self-attention (MHA) layer to model global dependencies. These additional layers are inserted after the encoder while maintaining the overall structure of the AE unchanged. The detailed configurations of AE, AE-C, and AE-A are illustrated in Table 3.

**Table 3:** Configuration of the autoencoder.

Layer composition	Input features	Output features	Additional parameters
Linear + BN + Dropout	Channels	1024	–
Linear + BN + Dropout	1024	512	–
Linear	512	256	–
(Only applied to AE-C and AE-A)			
Conv1D (AE-C only)	256	256	Kernel: 3, stride: 1, padding: 1
Multi-headAttention (AE-A only)	256	256	Heads: 8, embed dim: 256
Linear + BN + Dropout	256	512	–
Linear + BN + Dropout	512	1024	–
Linear	1024	Channels	–

To automate hyperparameter exploration and improve efficiency, we employ Grid Search, a technique from AutoML, to systematically search over specified hyperparameter values. We optimize the model using the Adam optimizer with an initial learning rate of $1\times 10^{-3}$. The batch size during training is set to 64, and early stopping is applied to prevent overfitting, capping the training at a maximum of 100 epochs. To ensure the model’s effectiveness, we validate its performance at the start, middle, and end of each epoch to monitor its performance and adjust the training process accordingly. The recursive input strategy is implemented with a window size of 5, a dynamic threshold ratio $\alpha$ of 0.005, and a patience level of 2. These parameters are determined empirically, based on observations of the datasets’ characteristics. The choice of a window size of 5 is motivated by the stability it provides in estimating error reduction trends, achieved by smoothing short-term fluctuations. The dynamic threshold ratio $\alpha$ of 0.005 ensures sensitivity to minor improvements, enabling effective detection of convergence, while a patience level of 2 balances responsiveness and robustness by tolerating minor fluctuations before halting recursion.

### Experimental results

**Table 4:** Comparison of RMSE (mean ± standard deviation) across different datasets with varying numbers of missing channels (*C*).

	Model	Dataset					Average
		ETTh1	ETTh2	ETTm1	ETTm2	Weather	
$C=1$	AE	0.492±0.003	0.541±0.006	0.474±0.002	0.450±0.002	0.898±0.040	0.571±0.018
	$\text {AE}_{\text {Re}}$	0.228±0.004	0.211±0.003	0.212±0.002	**0.232**±**0.002**	0.052±0.001	0.187±0.002
	AE-C	0.417±0.003	0.505±0.006	0.424±0.002	0.484±0.002	0.649±0.027	0.496±0.012
	$\text {AE-C}_{\text {Re}}$	0.183±0.003	**0.202**±**0.003**	0.213±0.002	0.237±0.002	0.048±0.001	0.177±0.002
	AE-A	0.412±0.002	0.462±0.005	0.422±0.002	0.476±0.002	0.890±0.038	0.532±0.017
	$\text {AE-A}_{\text {Re}}$	**0.175**±**0.002**	0.229±0.002	**0.173**±**0.002**	0.260±0.003	**0.046**±**0.001**	**0.176**±**0.002**
$C=2$	AE	0.802±0.006	0.953±0.006	0.775±0.002	0.775±0.002	1.789±0.033	1.018±0.015
	$\text {AE}_{\text {Re}}$	0.493±0.003	0.495±0.007	0.457±0.002	0.505±0.003	0.102±0.001	0.410±0.003
	AE-C	0.714±0.005	0.893±0.005	0.717±0.002	0.839±0.002	1.312±0.023	0.895±0.010
	$\text {AE-C}_{\text {Re}}$	0.444±0.004	0.492±0.004	0.474±0.002	**0.501**±**0.004**	0.411±0.026	0.464±0.012
	AE-A	0.694±0.004	0.824±0.004	0.707±0.002	0.835±0.002	1.771±0.032	0.966±0.014
	$\text {AE-A}_{\text {Re}}$	**0.425**±**0.006**	**0.458**±**0.006**	**0.395**±**0.002**	0.510±0.004	**0.086**±**0.000**	**0.374**±**0.004**
$C=3$	AE	1.073±0.004	1.278±0.006	1.026±0.001	1.031±0.003	2.677±0.049	1.417±0.022
	$\text {AE}_{\text {Re}}$	0.780±0.005	0.795±0.009	0.685±0.003	**0.738**±**0.004**	0.152±0.003	0.630±0.005
	AE-C	0.994±0.005	1.242±0.005	0.972±0.001	1.126±0.003	1.993±0.035	1.265±0.016
	$\text {AE-C}_{\text {Re}}$	0.736±0.003	0.897±0.008	0.714±0.002	0.768±0.005	1.122±0.052	0.847±0.023
	AE-A	0.974±0.005	1.150±0.005	0.972±0.001	1.142±0.003	2.647±0.047	1.377±0.021
	$\text {AE-A}_{\text {Re}}$	**0.698**±**0.007**	**0.679**±**0.005**	**0.632**±**0.002**	0.762±0.003	**0.121**±**0.001**	**0.578**±**0.004**

The results are averaged over 10 trials. AE denotes the standard autoencoder, $\text {AE}_{\text {Re}}$ represents the proposed autoencoder with a recursive input strategy and dynamic termination, AE-C and AE-A incorporate 1D CNN and multi-head Self-Attention (MHA), respectively, and $\text {AE-C}_{\text {Re}}$ and $\text {AE-A}_{\text {Re}}$ apply the recursive strategy to AE-C and AE-A. For each dataset and each number of missing channels, the best (minimum) values are indicated in bold.

**Fig. 3:**
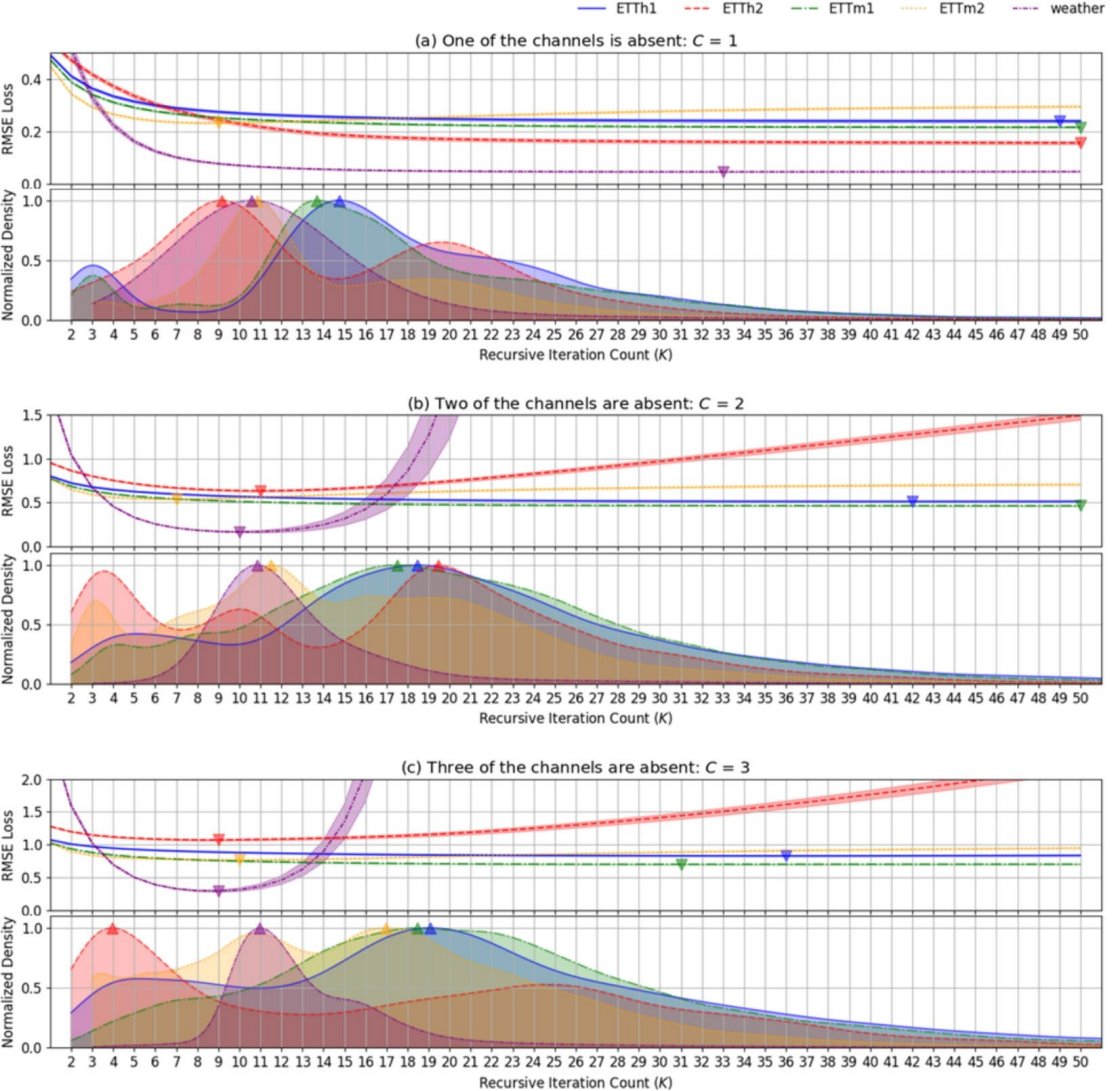
RMSE trends over iterations for AE (without dynamic termination) across different datasets and numbers of missing channels (*C*). The RMSE does not consistently decrease over iterations, and unnecessary iterations can lead to increased reconstruction errors or overfitting. Distribution of termination iterations for $\text {AE}_{\text {Re}}$ across different datasets. The algorithm dynamically determines the optimal number of iterations required for effective reconstruction, adapting to the data’s characteristics.

To evaluate the effectiveness of our proposed recursive input strategy with dynamic termination criteria, we conducted experiments comparing three approaches: the standard autoencoder (AE), which processes the input data once without recursive refinement; $\text {AE}_{\text {Fixed}}$, which employs recursive input with a fixed number of iterations but lacks dynamic termination; and our proposed $\text {AE}_{\text {Proposed}}$, which dynamically determines the optimal stopping point.

Missing data were generated through the random omission of values from one, two, or three channels, following the random points scenario described earlier. For each dataset and condition, 10 independent trials were conducted to account for variability in the random selection of missing channels and data points. Reconstruction performance was evaluated using the Root Mean Square Error (RMSE), with mean and standard deviation calculated across trials, as illustrated in Table 4.

The results indicate that the standard autoencoder (AE) struggles to reconstruct missing values accurately, particularly as the number of missing channels increases. This is expected, as AE learns a fixed mapping from input to output without explicit mechanisms to adapt to varying levels of missingness. The incorporation of 1D convolution (AE-C) and multi-head self-attention (AE-A) generally improves performance, suggesting that local feature extraction and global dependency modeling are beneficial for handling missing values. However, these enhancements are constrained, as the underlying reconstruction process remains deterministic and does not dynamically adjust based on the missing data structure.

In contrast, the proposed recursive input strategy ($\text {AE}_{\text {Re}}$) significantly enhances reconstruction performance across all datasets and missingness conditions. This improvement is particularly pronounced when the number of missing channels increases, highlighting the effectiveness of leveraging previously reconstructed values iteratively. As shown in Table 2, the recursive strategy consistently reduced reconstruction errors (RMSE), particularly noticeable as the number of missing channels (*C*) increased. For instance, on the ETTh1 dataset, the recursive model reduced RMSE by nearly 50% compared to the standard AE model. This result clearly supports our claim that iterative refinement significantly improves accuracy. Additionally, the integration of the recursive strategy into AE-C ($\text {AE-C}_{\text {Re}}$) and AE-A ($\text {AE-A}_{\text {Re}}$) further enhances performance, demonstrating that combining local feature extraction, global context modeling, and iterative refinement results in a more robust reconstruction framework.

The superior performance of $\text {AE-A}_{\text {Re}}$ over $\text {AE-C}_{\text {Re}}$ suggests that self-attention is particularly well-suited for capturing long-range dependencies among channels, which is crucial when multiple channels have missing values. Meanwhile, $\text {AE-C}_{\text {Re}}$ still outperforms its non-recursive counterpart, indicating that local feature extraction remains beneficial, especially in datasets where temporal or spatial correlations are prominent. The findings collectively demonstrate that the recursive input strategy, when combined with appropriate architectural modifications, effectively mitigates the limitations of standard autoencoders in missing data imputation.

To further assess the benefits of dynamic termination, a comparison was conducted between $\text {AE}_{\text {Re}}$ and $\text {AE}_{\text {Fixed}}$ using a fixed iteration count ($K_{max}$=100). $\text {AE}_{\text {Fixed}}$ initially reduces RMSE but eventually leads to its increase, highlighting the need for an optimal stopping point for accuracy and efficiency, as illustrated in Fig. 3. $\text {AE}_{\text {Re}}$ employs a dynamic termination mechanism that halts iterations appropriately when performance improvements become negligible or degrade, allowing the reconstruction process to remain effective and efficient. The combination of recursive refinement with dynamic termination enables $\text {AE}_{\text {Re}}$ to adapt effectively to diverse data characteristics and missing value scenarios, enhancing both accuracy and efficiency. Furthermore, the automation within dynamic termination increases the algorithm’s applicability across various datasets and conditions. The algorithm’s capacity to adjust iteration counts enables it to robustly handle diverse data distributions without the need for manual parameter tuning, making $\text {AE}_{\text {Re}}$ particularly suitable for real-world scenarios. This synergy between recursive refinement and dynamic termination yields consistent and significant performance gains, exploiting inter-channel correlations. These results underscore the adaptability and robustness of the proposed approach in addressing varying degrees of data loss. $\text {AE}_{\text {Re}}$ consistently delivers adaptable performance improvements across datasets and missing channel scenarios by enabling dynamic iteration adjustment and optimal stopping.

**Fig. 4:**
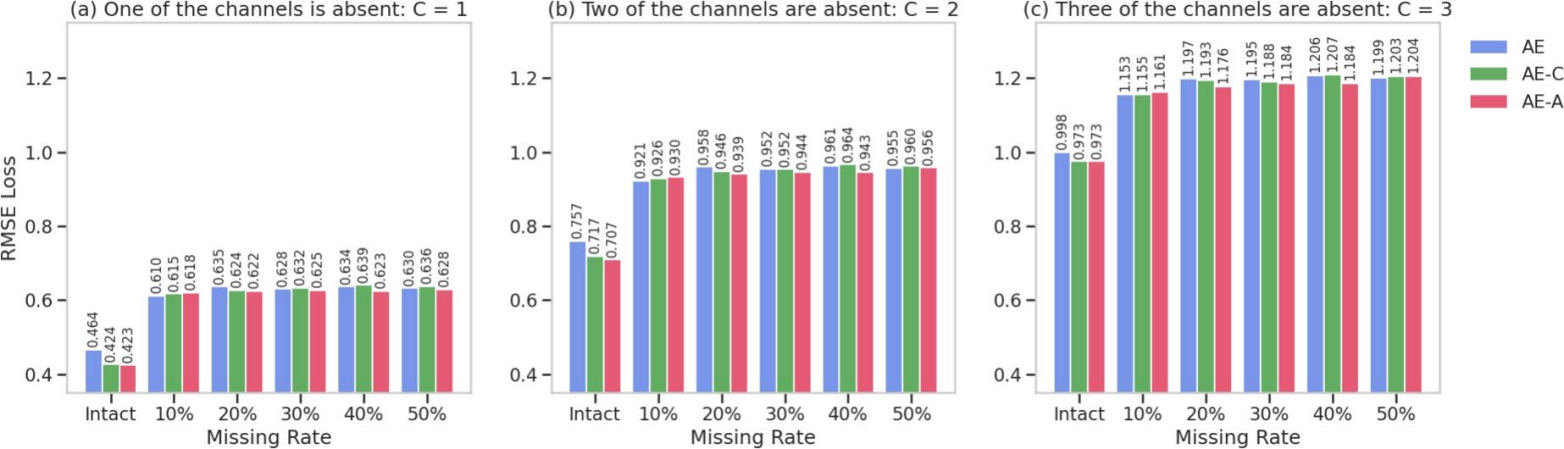
RMSE trends for models AE, AE-C, and AE-A, which were trained on intact data and data with 10 to 50% random missing rates. The performance of these models will be compared across scenarios with one to three missing channels (C).

In order to examine the impact of data completeness during training, experiments were conducted with AE, AE-C, and AE-A models trained on datasets with 0% (intact) to 50% missing data in 10% increments. Models trained with intact data captured the underlying patterns more effectively, resulting in superior reconstruction. As the missing rate increased, random omissions hindered the learning of intrinsic structures necessary for effective data reconstruction, leading to decreased performance. The proposed method, designed to train on normal data, functions independently of temporal dependencies and large dataset sizes, emphasizing the importance of data quality. This suggests that higher data validity and reliability are crucial for leveraging the proposed method to achieve optimal performance, as illustrated in Fig. 4.

## Discussion

The experimental results validate the effectiveness of the proposed data restoration algorithm in addressing missing sensor data across diverse application domains. By leveraging a recursive input strategy during inference, the algorithm iteratively refines the accuracy of restored values, addressing the limitations typically seen in conventional methods. Unlike traditional statistical approaches, which often struggle with nonlinear or multivariate dependencies, our method effectively reconstructs data even in complex interdependency scenarios. Additionally, compared to other deep learning-based methods, the recursive mechanism enables adaptive improvement without requiring extensive modifications to the model during training. One of the key advantages of the proposed method lies in its ability to operate without prior knowledge of missing data patterns. Unlike existing recursive techniques that rely on predefined configurations or labeled data, our approach dynamically adapts to various missing scenarios, enhancing its applicability in real-world environments. The dynamic stopping criterion can aid in ensuring computational efficiency and preventing overfitting, making the algorithm particularly suitable for applications that are time-sensitive or have limited resources, such as real-time monitoring in healthcare or autonomous systems.

However, the proposed method is not without limitations. While the dynamic stopping criterion effectively reduces computation, it may require fine-tuning to balance restoration accuracy and processing time in highly heterogeneous datasets. Additionally, the current approach assumes that the autoencoder is trained on complete datasets, which may not always be feasible in applications where only incomplete or corrupted data is available. To address these challenges, future research could explore lightweight variants of the autoencoder to improve real-time performance without sacrificing accuracy. Incorporating adaptive hyperparameter tuning could improve the model’s adaptability to different data sets by dynamically optimizing learning rates and the number of iterations. Furthermore, the integration of federated learning could support distributed sensor networks while preserving privacy. These explorations are critical to improving the model’s robustness and extending its applicability to more complex environments.

In summary, the proposed method demonstrates significant potential for improving the reliability of intelligent systems by effectively restoring missing sensor data. Its adaptability, accuracy, and efficiency make it a valuable tool for ensuring data integrity across diverse and complex applications.

## Conclusions

This study presents a novel autoencoder-based data restoration method incorporating a recursive input strategy and a dynamic stopping criterion. The recursive input strategy enables iterative refinement of missing data predictions, thereby significantly enhancing restoration accuracy. The dynamic stopping mechanism aids in ensuring computational efficiency by adaptively evaluating the point at which further iterations yield minimal improvements, suggesting the method’s potential utility in both real-time and large-scale applications. The experimental results demonstrate the robustness and versatility of the proposed method across a variety of datasets and missing data scenarios, including random and extensive data loss. Unlike traditional approaches, this method operates without requiring prior knowledge of missing data patterns or manual configuration, facilitating easy integration into existing systems. This automation improves the reliability and performance of sensor networks, making it advantageous for critical applications such as environmental monitoring, healthcare, and autonomous systems. Future work will focus on extending the proposed method to handle time-series datasets with strong temporal dependencies and exploring lightweight implementations for resource-limited environments. By addressing these challenges, the method can be further optimized for broader deployment, ensuring reliable and efficient data restoration in diverse domains.

## Data Availability

The datasets used in this paper are acquired at https://github.com/zhouhaoyi/ETDataset and https://www.bgc-jena.mpg.de/wetter/.
